# Redox Remodeling by Nutraceuticals for Prevention and Treatment of Acute and Chronic Inflammation

**DOI:** 10.3390/antiox12010132

**Published:** 2023-01-05

**Authors:** Claudia Petrarca, Davide Viola

**Affiliations:** 1 Department of Medicine and Aging Sciences, G. D’Annunzio University, 66100 Chieti, Italy; 2 Center for Advanced Studies and Technology, G. D’Annunzio University, 66100 Chieti, Italy

**Keywords:** antioxidant, ROS, immunomodulation, nutraceuticals, probiotic, postbiotic, diabetes

## Abstract

Antioxidant-rich dietary regimens are considered the best practice to maintain health, control inflammation, and prevent inflammatory diseases. Yet, nutraceuticals as food supplements are self-prescribed and purchasable over the counter by healthy individuals for the purpose of beneficial effects on fitness and aging. Hence, the effectiveness, safety, and correct intake of these compounds need to be better explored. Since redox-modulating activity of these compounds appears to be involved in activation and or suppression of immune cells, the preventive use of nutraceuticals is very attractive even for healthy people. This review focuses on redox- and immunomodulating nutraceuticals in the context of diabetes mellitus (DM). In fact, DM is an illustrative disease of latent and predictable inflammatory pathogenetic processes set out and sustained by oxidative stress. DM has been thoroughly investigated through in vitro and in vivo models. Furthermore, human DM is characterized by uncontrolled levels of glucose, a pivotal factor shaping immune responses. Hence, antioxidant nutraceuticals with multifaced activities, including glucose keeping, are described here. A greater number of such multi-player nutraceuticals might be identified using DM animal models and validated in clinical settings on genetic and environmental high-risk individuals.

## 1. Introduction

In Western countries a gradual change in native eating habits, mainly based on fresh and poorly processed foods, occurred through the second half of the twentieth century along with an overall socio-economic rise and globalization. In recent years, balanced nutrition has been established as a principle for healthy life [1]. Nevertheless, people’s eating habits still include excessive intake of sugars and fats. Consequently, they often display dysregulation of metabolism and cardiovascular function, known prodromes of chronic auto-inflammatory conditions. Food is regarded as a main environmental factor affecting differentiation and function of the immune system [2], and a diet naturally rich in antioxidant compounds—mainly based on edible vegetables and fruits—is considered the finest to maintain a healthful status and avoid getting diseases [3]. Indeed, nowadays antioxidant compounds are reputed “nutraceuticals”, i.e., food supplements providing a physiological advantage through a mechanism of action, for doses 2–10 fold higher than the recommended daily allowance [4]. However, some of these compounds perform as modulators of the redox potential of the surrounding milieu and are conditioned by it. Nevertheless, they are developed and marketed as key components of a balanced diet and as self-prescribed food supplements available over the counter. Such practice provides a larger intake than ever observed of these compounds for the purpose of beneficial effects for healthy individuals [5]. Furthermore, certain nutraceuticals appear to modulate the immune system, directly and/or indirectly. Recently, a large series of molecules and compounds present in food and plants has been discovered that may show interesting activities in humans [6]. Nonetheless, the guidelines of the American Diabetes Association do not provide any specific recommendation regarding the use of supplements or functional foods for diabetic patients while encouraging the consumption of products naturally rich in polyphenols and antioxidants [7]. Hence, the effectiveness, safety, and correct intake of these compounds need to be better explored. This review focuses on redox-modulating nutraceuticals in diabetes. This disease is paradigmatic of latent and predictable pathogenetic inflammatory processes representing an opportunity for preventive intervention with nutraceuticals. The beneficial and hazardous role of nutraceuticals in subclinical conditions sustained by activated or suppressed immunity is critically exposed and discussed.

## 2. Diabetes as an Archetypical Auto-Inflammatory Disease

Diabetes is an autoimmune disease characterized by damage and disruption of Langerhans’ β cells in the pancreas. These cells produce insulin, a peptide hormone crucially involved in keeping the glucose level in the blood within the physiological range (normal glycemia) by allowing receptor-mediated glucose uptake by cells and by inhibiting liver gluconeogenesis. Pancreatic β cells are intrinsically characterized by low scavenging antioxidant enzymes and cannot efficiently counteract oxidative stress [8]. In fact, unrestrained free radicals determine β cells’ destruction, through apoptosis, necrosis, and improper folding of their proteins including insulin. Macrophages are able to massively produce and release reactive oxygen species (ROS) and chemical mediators of inflammation. They are initiators of the subclinical inflammation process of pancreatic islets (insulites or, improperly, prediabetes), to which other cell types also participate. During that early phase, pancreatic (neo)antigens are exposed by professional antigen presenting cells (APC)—macrophages and dendritic cells (DC)—to specific T cells. Concertedly, DC and T cells activate specific antibody-producing B-cells, which had been likely primed in the course of antecedent responses to external pro-oxidant environmental factors. Thereof, they mount the acute cellular and humoral autoimmune response from which a detrimental chronic immune response might set off [9], if not controlled. Histological abnormalities of the pancreatic islets are diagnostic of full DM, although not handily in clinical practice and research. Instead, metabolic changes occur as early as two years before the acute onset of Type 1 diabetes (T1D) and are accompanied by a reduction of serum peptide c, a secondary product of the enzymatic conversion of inactive pro-insulin into active insulin. Loss of critical mass and histology of insulin-secreting cells, fasting hyperglycemia, and insulin resistance are secondary events to immune system activation and autoantibody generation [10,11]. T1D is determined by complex interactions between genetic and environmental factors through mediation of the innate and adaptive immune system leading to destruction of the pancreas and profound metabolic changes. Unmodifiable genetic signatures have been associated with a higher risk of T1D onset and development in siblings and monozygotic twins, and in subjects carrying definite human leukocyte antigen (HLA) involved self-antigen presentation (class I) or non-self-antigens (class II), as well as some non-HLA loci [11].

Remarkably, sub-clinical and clinical phases of both T1D and T2D can be evidenced by measuring well-assessed and interrelated biological markers, such as glycemia (glucose concentration in blood), and redox status. Indeed, in fasting conditions, glycemia over 100 mg/dL and up to 125 mg/dL, is a tag of early stages of disease (prediabetes) and is diagnostic of overt diabetes for values higher than 126 mg/dL. Moreover, after a loading curve test (oral glucose tolerance test) glycemia reaches very high levels diagnostic of reduced glucose tolerance in T2D for values >140 mg/dL or overt T1D for values over >200 mg/dL. Pathogenic surpluses of glucose in the blood are also detectable as glycated hemoglobin HbA1c >48 mmol/mol creatinine (>6.5%), another biomarker of diabetes [11].

One or more autoantibodies—namely anti-native insulin autoantibodies (IAA), islet cell antibodies (ICA), anti-glutamic acid decarboxylase antibodies (GADA), and anti-tyrosine phosphatase autoantibodies (IA-2A)—are generated early during the sub-clinical phase, even earlier than dysglycemia occurs [12]. They also represent accelerators of pathology and markers of its severity and progression. Notably, the higher the number of different circulating autoantibodies, the higher the relative risk in children, according to a 10-year long cohort study [13].

Type 2 diabetes (T2D) is a systemic condition called metabolic syndrome that is characterized by insulin resistance in adulthood, rather than destruction of β cells in childhood. Early T2D pathogenesis occurs in adipose tissue, where resident M1 macrophages become activated upon pro-oxidant stimuli and sustain local inflammation [14] and oxidative stress [15]. Afterward, the pancreas is reached by inflammatory mediators and ROS—generated in fat tissue—that cause islet β cell damage. However, the overall pancreatic function is maintained, and insulin is overproduced. Hyperinsulinemia downregulates glucose uptake producing the so-called insulin resistance through intracellular signaling pathways that are not yet completely understood. Meanwhile, endogenous synthesis of glucose by hepatocytes is restored and lipolysis is activated. Next, free fatty-acids are increased in blood, and adipose tissue is accumulated producing endocrine factors (leptin and resistin) that block carbohydrate metabolism [16]. Hyperglycemia develops when insulin secretion can no longer compensate for insulin resistance, originating a detrimental loop of insensitivity and dysfunction of the β cells. Notably, stimulation of the insulin receptor on T-cells potentiates their activity in inflammation and infection [17]. The pathogenetic changes last several years and are influenced by pro-inflammatory food regimens, sedentary lifestyle, and excess of visceral and abdominal fat. T2D is typically diagnosed in inactive and/or elderly people. Relative risk increases by age and is most prevalent in adults (>90%) and over-65 patients (>33%). T2D is also epidemically increasing among obese children [18]. Insulin resistance is also observed in low-birth-weight children of obese mothers. Gestational diabetes (diagnosed for the first-time during pregnancy) occurs more frequently in overweight, hyper-insulinemic, insulin-resistant or lean insulin-deficient women. These women are subjects at high risk of developing T2D in life [18].

Recently, Severe Acute Respiratory Syndrome-Coronavirus 2 (SARS-CoV-2) infection has been shown to impair insulin/insulin growth factor (IGF) signaling pathway genes in the host pancreatic cells attributed to interferon regulatory factor 1 (IRF1) [19]. This finding sustains the hypothesis of the viral origin of DM and also shows that infected people might be at higher risk of developing DM.

Glucose and oxidative stress are determinants of the immune response signature in these inflammatory diseases, as described next.

## 3. Experimental Study Models of DM in the Context of Redox and Immunomodulatory Effects of Nutraceuticals

In vivo models (two mouse models and one rat model) have been developed that well recapitulate the pathogenesis and pathology characteristics of DM: the NOD (non-obese diabetes—genetically engineered) mouse model and the pharmacologically-induced obese rat model, widely used to elucidate mechanisms and test drugs. NOD recapitulate the immunopathology of human T1D as autoantigen recognition by major histocompatibility (MHC) II variants affecting the presentation of islet-derived antigen to T cells. Hence, it is also a model of prediabetic condition. NOD has revealed the role of environmental factors in the prevention and development of this disease [20]. The second model is induced by streptozotocin (STZ), an indirect DNA-alkylating compound acting via xanthine oxidase. Fast induction of T1D is achieved in STZ-induced mice fed with a high-fat diet totaling 37% of daily caloric intake [21]. Notably, both murine models are characterized by increased oxidized oxygen and nitrogen species. Immunoregulatory properties of pro/postbiotics and nutraceuticals in diabetes have been investigated mainly in vitro and in pre-clinical studies using these models.

A third most used model recapitulates the physiopathology of T2D, which can be induced in healthy rats by feeding them with a high-fat diet and sucrose for two weeks [22]. In addition, obese mice are used to study insulin sensitivity and resistance [23].

Extremely heterogeneous cell lines are used as in vitro study models. Among these, there are primary peripheral blood (PB)-derived human macrophages, neutrophils, lymphocytes, and human umbilical vascular endothelial cells (HUVEC). Moreover, a varied panel of established cell lines has been examined, such as Henrietta Lacks’ (HeLa) cervical carcinoma, human interleukin-2 (IL-2)-dependent natural killer (NK)92, macrophage-like, Abelson leukemia virus-transformed cell line derived from BALB/c mice (RAW), C57/BL6 murine immortalized microglial BV-2, murine monocyte-like U937, and human immortalized T cells leukemia Jurkat.

## 4. M1 Macrophages Initiate and Generate Oxidative Stress and Cell Damage

Endogenous free radicals of oxygen are produced as unavoidable side products of oxidative phosphorylation (OXPHOS) for de novo biosynthesis of long-chain fatty acids (FAS). Recently, endogenous ROS have been proposed as regulators of immune cell function, controlling their differentiation and proliferation. Indeed, different functional subsets of innate cells appear to be conditioned by extracellular and intracellular oxidative stress, a hallmark of inflammation, and display distinct metabolic profiles. M1 Macrophages are involved in inflammatory response to eliminate environmental offenses, such as viruses and pollutants, primarily by releasing ROS and subsequently by phagocytic activity and degradation. Free radicals can directly promote the activation of redox-dependent transcription factor NF-κB. In macrophages, this mechanism leads to the production and release of crucial pro-inflammatory cytokines, interleukin (IL)-1β, IL-6, and tumor necrosis factor (TNF)-α. The high energy demand to accomplish these activities relies on the aerobic glycolytic pathway in normal oxygen conditions. Any imbalance of intracellular ROS and the phagocytic activity itself leads to the release of ROS in the extracellular milieu, thereby conditioning the activity of other bystander cells. Free radicals induce inflammation, cytolysis, and apoptosis which constitute the complete signal for activation of autoreactive T-cells. Ultimately, such alteration of the redox potential is considered a factor promoting diabetes. Indeed, in pancreatic islets, oxidative stress activates the nucleotide-binding domain and leucine-rich repeat (NLR) pyrin domain containing 3 (P3) (NLRP3) (via release of the redox-sensitive inhibitor of inflammasome activator TRX1, TXNIP) that cleaves pro-inflammatory IL-1β precursor into its active form. Mitochondrial (DNA) oxidation products can do so, too. The activation of redox-sensitive transcription factor α (HIF-1α) initiates the third step of expression of pro-inflammatory cytokines IL-1β and TNF-α that, in turn, positively controls glycolysis amplifying M1 activation and ROS production. ROS can also stimulate IL-1β production through the biosynthesis and stabilization of GSH (from serine-derived glycine). In low oxygen conditions, M1 expresses inducible nitric oxide synthase (iNOS) and produces high levels of nitric oxide (NO) and reactive nitrogen species (RNS). Furthermore, NO can downmodulate the expression of transcription factor Foxp3, which is specific for induction of regulatory T cells (Treg) and lessen priming of autoantigen-specific Treg [24]. Under specific conditions, M2 cells can revert this scenario. In fact, M2 are important players in inflammation resolution and healing processes, also exerting an inhibitory activity on M1 cells. They function mainly through the low oxygen-requiring pathway, OXPHOS, and produce low ROS. Anti-inflammatory myeloid-derived suppressor cells (MD-SC), from which pro-inflammatory M1 cells also derive, diverge from them for metabolic activity and ROS profiles, such as resting macrophages [25]. In healthy individuals, MD-SC differentiate into pro-tolerogenic regulatory DC (DCreg) secreting IL-10, under the stimulus of GM-CSF growth factor. If prompted by G-CSF, they become DC2 which prime differentiation and activation of Th2 cells and are able to contrast Th1 sustaining harmful inflammatory response to autoantigens [26] (Table 1).

## 5. Glucose and Oxidative Stress Determine the Metabolic Signature of T Cell Function

Following activation and differentiation, pro-inflammatory T cells are distinguished from anti-inflammatory Treg based on their metabolic signatures. Distinct metabolic programs control the development of an effective and balanced immune response determined by the differentiation status (naïve or memory) and effector profile (Th1, Th17, or Treg).

**Figure 1:**
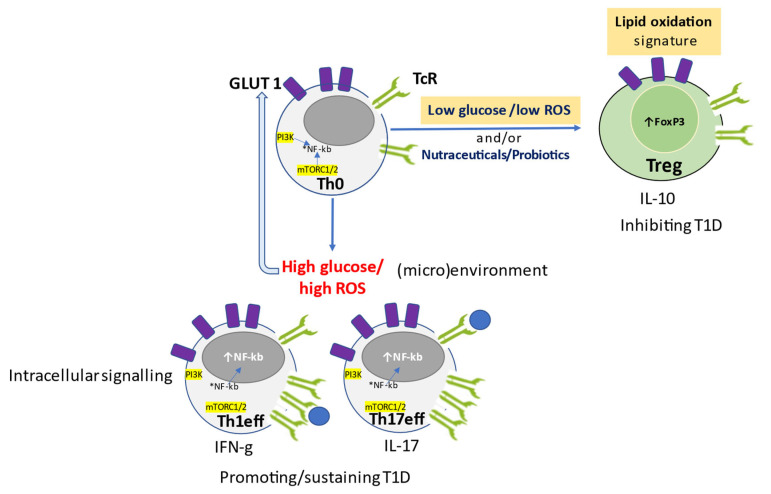
ROS-oriented immunometabolism proliferation and differentiation of T cells.

Glucose controls the development of an effective and balanced immune response. Anaerobic glycolysis regulates glucose uptake, activation, and differentiation of CD4+ T cells. Inflammatory Th1 and autoimmune Th17 cells, which share some metabolic regulators, are energetically sustained by high glycolytic activity. Instead, Treg cells, which are regulated differently, do not need anaerobic glycolysis to work. Indeed, the glycolysis inhibitor rapamycin promotes the generation of functional Tregs in vitro, preventing allograft rejection in vivo. Interestingly, the negative regulation of thioredoxin system (TRX) 1, through inhibitor TXNIP, restrains Teff cell proliferation and inhibits glucose transporter 1 (GLUT1) expression, hence hampering glucose metabolism. Glucose uptake and aerobic glycolysis are restored in Teff upon engagement of the immune synapse, CD28 and TCR, and downmodulation of TXNIP (Figure 1) [2,27]. GLUT1 expression and glycolysis increase in response to ROS through NF-κB signaling. Insulin receptor (INSR)-deficient T cells cannot mount an anti-viral response in vivo, but stimulation of the INSR boosts T-cell immunity in inflammation and infection [18] (Figure 1).

Naïve T cells (Th0), the precursors of functional T cells, are quiescent and non-biosynthetic cells receiving energy supply and maintaining redox homeostasis based on OXPHOS. Extracellular low redox potential (high GSH:GSSG ratio) and reducing conditions (APC-derived cystine, Cys2) are activation signals for naïve T cells that differentiate into T cells requiring anaerobic glycolysis, low level of intracellular ROS maintained by electron transport chain (ETC), and pentose phosphate pathway (PPP) for fatty acid synthesis (FAS) to sustain cell growth and maintain redox homeostasis. They become activated when intracellular reducing conditions occur. In effect, ROS, reduced nicotinamide adenine dinucleotide phosphate (NADPH), and thioredoxin (TRX), promote proliferation of T cells which upregulate IL-2 receptors, and activate pro-inflammatory transcription factor NF-κB and NLRP3 inflammasome. Instead, fatty acid oxidation (FAO) is critical to promote (proliferating) memory T cells (Figure 1, Table 1).

**Figure 2:**
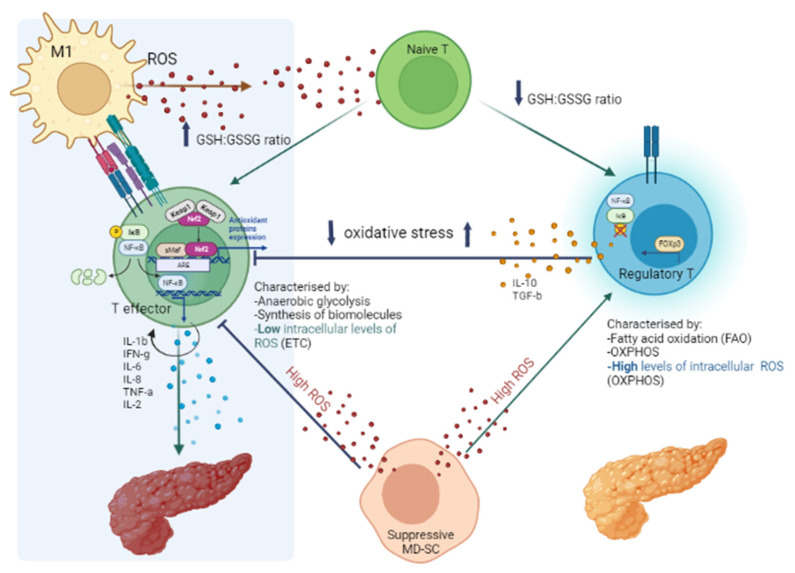
Redox remodeling mechanism in immune-mediated inflammatory and inhibitory responses.

Oxidative stress is required for the modulation of T cell effector functions. In fact, T cells have ROS-sensor/binding nuclear transcription factors HIF-1α and Iκ kinase (IκK), directly controlling cytokine gene expression, and Kelch like ECH-associated protein 1 (KEAP-1)-controlling antioxidant enzyme expression. HIF-1α is crucial for the commitment of naïve cells toward Treg or Teff cell programs, respectively, depending on its low or high expression. Its upregulation also favors Th17 differentiation through increased transcription factor RAR-related orphan receptor gamma (ROR-γ), as well as the glycolytic shift of macrophage and NF-κB activation in low oxygen conditions. Instead, T regulatory cells are suppressed by HIF-1α. A high redox potential environment triggers naïve T cells to become regulatory T cells characterized by both oxygen-dependent FAO and OXPHOS (Figure 2), producing high amounts of ROS able to stabilize their specific transcription factor, FoxP3, if HIF-1α is low (Figure 1, Table 1). It appears likely that subsets of adaptative immune cells can be reprogrammed by antioxidant and NF-κB inhibiting substances to acquire anti-inflammatory properties [28]. Redox components of the thioredoxin (TRX) and glutathione (GSH) systems, as well as the transcription factor NRF2, are crucial regulators of intracellular redox potential and lineage commitment of immune cells. Recently, endogenous ROS has been proposed as a regulator of differentiation and proliferation of immune cells. This condition might arise from de novo biosynthesis of long-chain fatty acids (FAS) during which endogenous free radicals of oxygen are generated as unavoidable side products of oxidative phosphorylation (OXPHOS). ROS, TRX, and NADPH, produced through the pentose phosphate pathway (PPP), maintain redox homeostasis and promote inflammation sustained by NF-κB and NLRP3 inflammasome responses, which are essential for the proliferation of activated effector T cells. Instead, fatty acid oxidation (FAO) is critical to promote functional memory cells, which, in turn, are sustained by Teff which upregulate IL-2R. Meanwhile, NADPH is the upstream electron donor for thioredoxin (non-oxidative dNTP biosynthesis) and GSH (ROS scavenger), peroxiredoxins (hydrogen peroxide scavengers), glutaredoxins (GRX), and glutathione peroxidase.

The GSH system quenches/scavenges ROS, supports IL-1β expression in macrophages, primes Teff cells, and prevents ferroptosis of CD8+ memory T cells, whereas it limits serine metabolism in inhibitory Treg cells. Heightened metabolic activity in activated T cells results in ROS production providing the so-called “third signal” for full activation of T cells. However, excessive and/or prolonged ROS signaling in T cells results in altered metabolic pathways and impaired inflammatory responses.

Scavenging ROS during naïve T cell activation blocks terminal differentiation and favors the generation and persistence of long-lived memory T cells with stem-cell like properties.

Indeed, low levels of ROS originating from ETCs/OXPHOS are required for the nuclear factor of activated T cell (NFAT) activation and subsequent IL-2/IL-4 production and antigen-specific Theff activation and proliferation. Hence, the extracellular redox potential determines the differentiation lineage of immune cells, each having a distinctive functional property mediated by the production of a defined subset of cytokines [29]. Notably, once the immune cell subset commitment has been established, its functional behavior is not easily tunable or switched off by external interventions since, at that point, cells work with their own metabolic pathway/endogenous ROS and become resistant to extracellular redox conditions. As an example, autoimmune actively proliferating CD4^+^ T cells from patients with rheumatoid arthritis (RA), use glucose and the Pentose Phosphate Pathway (PPP) to generate intracellular reducing conditions (NADPH) and low ROS levels that keep the inflammatory condition going. However, ROS have been shown to possess anti-inflammatory activity in a model of human synovitis [30] (Table 1).

Immune cells can maintain the intracellular redox homeostasis through the glutathione antioxidant system. Reduced glutathione (GSH) is synthetized starting from two unreactive amino acid precursors, cysteine (Cys) and glutamine (Glu). They are conditionally essential amino acids in the human diet that are obtained through the catabolism of dietary proteins, requiring pro-oxidant extracellular milieu. They can also be produced endogenously by the hepatic metabolism from homocystein and glutamine-cysteine as substrates. Extracellularly released Cys and Glu can then be taken up by cells expressing specific antiporter systems. T cells have similar antiporter systems allowing uptake of the two GSH precursors as such and up-modulation of the transporters is associated with T cell proliferation and effector function, positively controlled by mammalian target of rapamycin complex 1 (mTORC1) and proto-oncogene c-MYC [31].

Activation and proliferation of Theff cells need a reducing milieu in the immune synapse that is supported by antigen presenting cells, especially dendritic cells [32]. Instead, Treg miss a functional antiporter system for Cys uptake. In gut-associated Treg, the inhibitory function of Teff depends on a reducing microenvironment supported by professional antigen-presenting cells (APC) able to synthetize and release Cys. In addition, Treg can inhibit the secretion of GSH by DCs and suppress extracellular Cys accumulation. In this way, they generate a higher redox potential which is suppressive for T-cell proliferation. Such redox remodeling mechanism functions only in Treg (and in naïve T cells), but not in pro-inflammatory Theff and macrophages [33] (Figure 2).

Lineage commitment of the diverse subsets of immune cells relies on nuclear factor erythroid 2–related factor 2 (Nrf2), the main transcription regulator of antioxidant/cytoprotective enzyme genes (catalase, glutamate-cysteine ligase, glutathione peroxidase, and superoxide dismutase). Nrf2 is a redox sensor maintained in its cytosolic inactive form by binding to KEAP1 inhibitor. In case of cytoplasmic oxidative stress increase, Nrf2 dissociates from its (phosphorylated) inhibitor and translocates into the nucleus, where it heterodimerizes with sMAF, and binds antioxidant response elements (ARE) to initiate the transcription of those genes (Figure 2). In immune cells, the role of activated Nrf2 is multifaceted, depending on the redox potential: in normal conditions, it inhibits prolonged activation of pro-inflammatory M1 through downmodulation of pro-inflammatory cytokine genes (RNA pol II) and interferes with NF-κB translocation. In the presence of high ROS, it is critical for the engagement and/or sustainment of M1 macrophages. In low ROS conditions, it can activate M2 macrophages to produce anti-inflammatory metabolites (such as itaconate and prostaglandins). Furthermore, in multiple sclerosis, induction of Nrf2 promotes Treg cell differentiation and survival and reduction of symptoms. In tumor settings, activated Nrf2 interferes with Th1eff cells and M1 activation of NLRP3 inflammasome and pro-inflammatory cytokine genes, conferring cytoprotection to tumor cells.

## 6. Role of Nutraceuticals in Redox-Remodeling, Anti-Inflammatory Response, and Glucose Control to Prevent Diabetes

Microbial nutraceuticals are continuously produced by human intestinal microbiota as a critical player in the complex and multifactorial mechanism that leads to T1D and other inflammatory autoimmune diseases. It consists of a set of symbiotic microorganisms that include thousands of billion probiotic bacteria, principally belonging to the *Firmicutes* and *Bacteroidetes phyla* inherited from the mother during natural delivery, has the foremost role of shaping and balancing the immune system and contributing to redox homeostasis, also providing nutritional metabolites, such as postbiotic vitamins and butyrate. In healthy people, such dynamic and balanced microbic flora adjuvate the immune system to maintain tolerance and suppress inflammatory and autoimmune responses. The microbiota can deteriorate into imbalanced dysbiosis triggered by environmental factors, comprising foods, and promoting inflammatory conditions such as insulitis. The early dysbiosis events damage the intestinal epithelium. Consequently, the abnormal passage of microbial and non-microbial antigens can occur resulting in local increase of antigen trafficking and intestinal inflammation in the gut-associated lymphoid tissue (GALT). Inflammation can further lead to the onset of autoreactive systemic responses, through the blood flow. For instance, intestinal infection sustained by *Clostridium rodentium*, able to demolish the intestinal epithelium, anticipates its appearance of genetically determined insulitis [34] and favors activation of autoreactive T cells of the gut mucosa [35]. Instead, intestinal colonization with probiotic *Akkermansia muciniphila* counteracts autoimmune diabetes by increasing the secretion of antibacterial peptides and mucus production [36]. A large and timely reduction of the incidence of T1D was also observed when a combination of *Lactobacilli* (*acidophilus*, *casei*, *reuteri*), *Bifidobacterium bifidium* and *Streptococcus thermophilus* (IRT5), referable to induction of Treg and concomitant reduction of Th1 polarization also associated to substantial tissue repair (i.e., reduced intestinal permeability and insulitis, and increase of β cells mass) [37]. Following these observations, eubiotic microbial strains have been investigated for potential preventive and protective roles in T1D models. Among these, the most studied are *Lactobacilli, Bifidobacteria* and certain *Clostridia* producing short-chain fatty acids (SCFAs), side products of the dietary fiber intestinal fermentation by these eubiotic strains. In fact, these postbiotic fatty acids are relevant to the maintenance of intestinal immune homeostasis and reduce chronic inflammation. Acetate and butyrate are involved in immunomodulation through different and partly synergistic mechanisms. A diet rich in acetate is associated with loss of antigen presentation function by competent B cells, which become unable to stimulate autoreactive T cells. In addition, butyrate-rich diet up-modulates occludine, reinforces mucosal tight-junctions, and promotes the expansion of regulatory T cells, which are protective against (auto)immune reactions [38]. Furthermore, butyrate prevents the onset of diabetes triggering the differentiation of regulatory T cells (Tregs) able to inhibit inflammatory responses mediated by the Th1 effector cells and their cytokine interferon (IFN)-γ, at both intestinal and systemic sites [39]. Indeed, intestinal fermentation supported by *Bacteroides* determined the increase of these postbiotics in the peripheral blood associated with a reduction of insulitis [34]. Furthermore, *B. fragilis*, through its surface polysaccharide A, stimulates the production of the inhibitory cytokine IL-10, produced by Treg cells, and suppresses the responses of Th17 cells, sustaining autoimmune responses [20]. Another study confirmed that acetate and butyrate, provided as a naturally rich diet, are associated with downmodulation of autoreactive T cells and, as a food supplement, reduce the incidence of T1D [40]. Human studies have shown that initiation of the autoimmune process is associated with alterations in the structure of mucus and/or increased permeability of the intestinal epithelium [41]. Clinical case-control studies in patients affected by T1D have shown that duodenal mucosa is characterized by alterations of the microbiota and by pro-inflammatory immunological profile [42]. Inversely, specific probiotic strains (*Firmicutes, Lactobacilli*, and *Bifidobacteria*) able to induce Treg cells and promote immunological tolerance to self-antigens are underrepresented [43]. Furthermore, in children at higher genetic risk of T1D, early probiotic supplementation is associated with a reduced risk of islet autoimmunity and, for a peculiar genetic trait, a causal relationship between probiotic supplementation and reduced risk was established [13] (Table 2).

Plant nutraceuticals are principally known for their antioxidant activity. Among them, polyphenols, triterpenes, fatty acids, phytochemicals, and postbiotic compounds act through the radical scavenging mechanism that instantly removes reactive oxygen and nitrogen species. Therefore, degradation of biomolecules and formation of additional free radicals are limited [85]. Other recent studies show that certain antioxidant nutraceuticals are also anti-inflammatory. In fact, they can inhibit the transcription of NF-κB (by direct binding or by blocking the phosphorylation of the inhibitory protein I-κK), and mitogen-activated protein (MAP)-kinases, involved in the expression of pro-inflammatory cytokines TNF-α and IL-12. Furthermore, various polyphenols can inhibit the inflammatory pathway controlled by ciclo-/lipo-oxygenase (COX/LOX) family members, in accordance with their structural homology to non-steroidal anti-inflammatory drugs (NSAIDs). Polyphenols (curcumin, quercetin, epigallocatechin gallate—EGCG), isothiocyanates (allicin), stilbenes (resveratrol) and flavonoids (aspalathin), have been suggested to be cytoprotective in cellular and animal models. Since their protection is lost when Nrf2 is experimentally inactivated or deleted, this signaling pathway is likely to be involved in the antioxidant activity highly present in coffee, broccoli, beetroot, berries, pomegranate, curcuma, and cocoa [59]. Curcumin (*Curcuma longa*) is a natural phenolic orange-yellow compound found at high concentrations only in the turmeric spice contained in the roots of Zingiberaceae plants, to which ginger belongs. It is barely found in natural foods, but it is widely used as dietary supplement called curry for food coloring (E100) and flavoring, mostly in the south-east, and as Ayurvedic medicine, too. Curcumin showed antioxidant and anti-inflammatory properties in obesity and diabetes favoring eubiotic microorganism growth and gut healing, through microRNA modulation [45]. Curcumin has been shown to exhibit therapeutic potential also in other chronic illnesses in which inflammation plays a major role. It has been investigated on a mouse model of Alzheimer’s disease induced by heavy metals, hence high ROS, in the brain. Curcumin reduced levels of beta amyloid aggregation and oxidized proteins and prevented cognitive deficits. In fact, two curcumin molecules can bind redox active metals Cu^2+^ or Fe^2+^ ions. Hence, chelation of metals is a likely mechanism for curcumin to reduce oxidative neurotoxicity and amyloid aggregation. In addition, curcumin has been proposed to suppress inflammatory damage by preventing metal induction of NF-κB [80]. Moreover, it is able to induce the expression and secretion of the inhibitory cytokine IL-10 in vitro and in preclinical models to enhance its action in many tissues and also to modulate the pathophysiology of inflammatory diseases (i.e., allergy). Notably, it also affects the response to infections and cancer through its effect on the secretion of IL-10. The immunomodulatory properties of curcumin deserve further study for its application in the treatment of immune system pathologies, such as T1D [79]. Methanolic oregano extract (MOE), a concentrate of polyphenols, reduced the incidence of diabetes and preserved insulin secretion in pre-diabetic NOD mice. In addition, MOE eliminated reactive oxygen and nitrogen species. MOE treatment alleviated Th17-mediated proinflammatory response and enhanced Th2 and Treg-mediated anti-inflammatory effect by impacting specific signaling pathways and transcription factors. Importantly, MOE preserved β cells from apoptosis in vitro as well as mice from developing diabetes. Likely, more than one substance is present in MOE providing antioxidant, immunomodulator and anti-apoptotic activities [47]. Epigallocatechin gallate (ECG) and epicatechin (EC), two plentiful polyphenols in green tea, were evaluated as anti-diabetic compounds. Treatment with ECG/EC 0.05% in drinking water appeared to prevent loss of β cell mass, increase insulin levels, decrease hemoglobin A1C levels, and lead to overall improvement of insulitis. The latter finding is explained by the immune-modulating functions of these compounds. In fact, they could downmodulate NF-κB and its downstream pro-inflammatory cytokines IL-1β, IL-6, TNF-α, and IFN-γ, as well as TRL-4, COX II, while promoting higher production of the inhibitory cytokine IL-10 [86]. Ursolic acid (UA), a pentacyclic triterpene hydroxy acid (3β-hydroxyurs-12-en-28-oic acid), forms aglycone with free acid or triterpenoid saponins. It is present in the human diet because of its widespread availability as epicuticular wax found in fruit peels. It is most abundant in Malus pumila (apple), Vaccimum spp. (blueberries), Vaccinium macrocarpon (cranberry), Olea europaea (olive), as well as in spicy herbs such as Ocimum basilicum (basil), Origanum vulgare, Rosmarinus officinalis, Salvia, and Thymus (thyme). In diabetic STZ mice, UA significantly reduces blood glucose levels and preserves insulin clusters within pancreatic β-cells in treated diabetic mice compared to the diabetic not treated (anti-diabetic), through an unknown mechanism. In addition, UA causes unresponsiveness in vitro of T cells from those mice even if stimulated with the polyclonal activator Concanavalin A (ConA), a mannose/glucose-binding lectin used in lymphocytes proliferation tests, as well as the associated cytokine production [87]. These deficits were recovered by UA-treatment (immunomodulatory function) [21]. Aronia melanocarpa, or chokeberry, is a plant producing berries that are particularly valued for their content in polyphenols, such as phenolic acids (neochlorogenic and chlorogenic acids), flavonoids (anthocyanins, proanthocyanidins and flavonols) and cyanidins (cyanidin-3-galactoside, cyanidin-3-arabinoside), and epicatechin. Aronia has anti-inflammatory properties on human monocytes. Specifically, it inhibits the activation of NF-κB, and consequently blocks the release of pro-inflammatory cytokines IL-6, IL-8 and TNF-α, as well as prostaglandin E2 (PGE2) formation [88]. These bioactive compounds also confer antioxidant and antidiabetic properties to this blackberry that effectively improve glucose metabolism controlling postprandial hyperglycemia, through the inhibition of α-glucosidase and α-amylase enzymes. Streptozotocin induced diabetic mice studies confirmed that oral administration of Aronia 100 mg/kg was associated with lower increase of glycemia and a protective effect against β cells compared to the diabetic control group not treated with Aronia [48]. One clinical trial showed that Aronia stabilizes carbohydrate metabolism after at least three months of daily consumption of 200 mL juice [50]. Recently, small adenosine-based non-peptidyl compounds, typical of plants and fungi, can directly act as ROS scavengers and have been found in vitro and in vivo to mimic the activity of insulin and pro-tolerogenic growth factors promoting DCreg differentiation [26,72]. Flavonoids from several vegetal sources could inhibit Th17 cells and stimulate Treg cells in experimental RA [89]. Despite the low number of studies performed to date, they all show a potential application of these compounds in humans [2,27,90,91]. The potentially preventive effects of nutraceuticals in human diabetes described here are summarized in Table 2.

## 7. Conclusions

In this review, we focused on auto-inflammatory diseases characterized by long latency and early biological markers that offer an opportunity for preventive interventions and status monitoring.

In fact, human DM is extensively studied since cellular and murine models are available for mechanistic and physiopathology studies. Moreover, susceptibility to developing DM is highly predictable at early stages by evaluating genetic factors, autoantibodies, and oxidative stress, which represent a main trigger [15].

So far, investigations on nutraceuticals conducted in disease settings have shown exciting results and suggest that they might be attractive even for healthy people.

Nowadays, the available chronic therapeutic treatments can slow the disease worsening, but they still have a substantial impact on patients’ quality of life.

Despite the innumerable nutraceuticals that have been studied at different levels of investigation, from experimental models to epidemiological studies, there are no clinical studies that might incontrovertibly sustain them as a preventive treatment of inflammatory diseases. Nevertheless, a large self-prescribed consumption is observed also among healthy subjects.

Recent findings on the role of oxidative stress point out the redox remodeling to control detrimental immune responses and thwart and shut down innate and adaptive autoimmune effector cells directed against pancreatic islet β cells.

At this scope, we performed a PubMed search to select original articles and recent high-impact reviews on antioxidant nutraceuticals in diabetes able to usefully modulate glycemia, and/or oxidative stress and/or immune cell and cytokine profiles. A series of twenty-two nutraceuticals have been retrieved with one or more of these biological characteristics (Table 2). Particularly, these nutraceuticals appear beneficial against DM, where the inflammatory component plays a fundamental role, and would be worthy of further investigation in humans. However, other anti-diabetic antioxidant nutraceuticals potentially promote detrimental pro-inflammatory responses by stimulating M1, effector Th1 cells, antibodies, and proinflammatory cytokines in DM models (Table 2).

Furthermore, such interventions on redox and immune dyshomeostasis might be detrimental in disease settings for which high redox potential and pro-inflammatory immunity are auspicial, such as anti-tumor responses. Instead, five nutraceuticals were found to be pro-inflammatory and able to trigger IL-1β-mediated signaling. In a recent review, we highlighted that phytochemicals with antioxidant activity provide a synergistic effect to induce oxidative stress and apoptosis/autophagy of malignant multiple myeloma (MM) cells when combined with the canonical anti-proteosome drug [92].

Other studies have focused on antioxidants targeting the critical ROS-producing organelles (mitochondria) as potential therapeutic agents to restore normal physiology and redox homeostasis in inflammatory diseases [93]. However, the nutraceuticals described here appear to be more than “just” antioxidants and seemingly suitable to overcome the issue of possible weakening of protective Th1-mediated immune responses, but still could adversely promote smoldering and indolent MM or T2D [94].

Clinical research focusing on the described nutraceuticals might clarify their preventive use in healthy individuals. The current options for DM treatment and associated conditions are expensive, life-threatening, and potentially toxic [95]. Hence, studies on nutraceuticals as adjuvant compounds to improve efficacy and lower side effects are worthwhile. Nutraceuticals, supplements, and functional foods deserve further studies either in subjects at high risk of DM or in full-blown patients upon precise redox status, immunological assessments [96,97], and nutritional intake [98].

**Figure 3:**
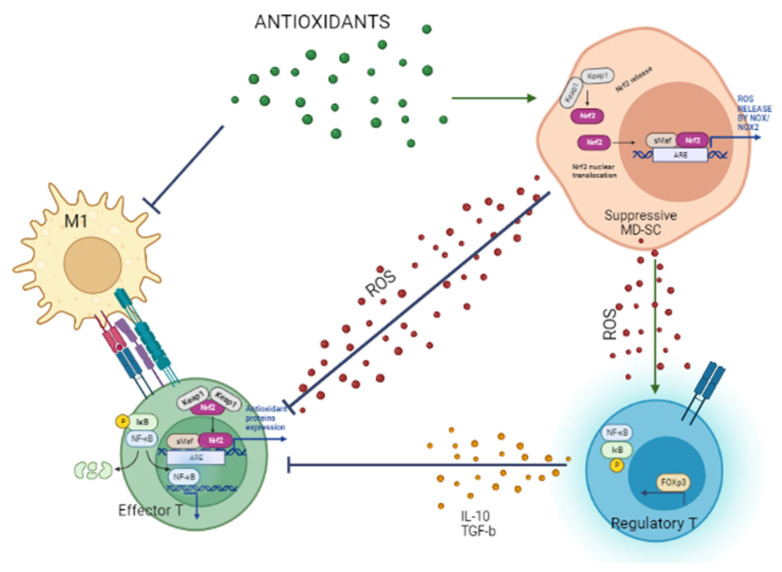
Potential immunomodulating role of a nutraceutical as an antioxidant.

In conclusion, the present review describes several “antioxidant” nutraceuticals as potential glucose-keepers and redox modulators of immune cells in (pre-)DM leading to inhibition/suppression of pro-inflammatory macrophages and Th cells (Figure 3).

Neutraceuticals are variously found in natural edible sources or can be extracted and concentrated from them as formulable supplements. Among these are plant leaves (tea, oregano, olive oil), whole fruits (grapes, olives, mango), beans (coffee, soy) and berries (berberis, aronia), legumes, and animal products (fish and meat). Nutraceuticals described here could be preventives as nutraceutical-rich food regimens, supplements, and/or combination with disease-specific drugs and treatments.

## Figures and Tables

**Table 1:** Redox parameters of immune cells experimentally tunable by nutraceuticals.

Immune Cell Type	Subset	Metabolic Pathway	Redox Potential (Extracellular)	Redox Potential (Intracellular)	ROS-Sensor Genes	Antioxidant Enzymes	Nutraceuticals
MACROPHAGES (innate immunity)	M1	GLYCOLYSIS (AEROBIC) FAS PPP OXPHOS	Inflammation/ ROS	High RNS	HIF-1α NLR-P3 NRF-2 TRX-1	CATALASE GSH SOD	ISOFLAVONS MANGIFERIN MARESIN-1 § PUFA SAPONINS SFN # SQUALENE β-SYTOSTEROL *
	M2	FAO OXPHOS	No influence	No free radicals	Itaconate (anti-inflammatory metabolite)	None	MARESIN-1 § SAFFRON SFN # β-SYTOSTEROL *
T CELLS (adaptive immunity)	NAIVE (Th0)	OXPHOS	Quiescent status				Not described, yet
	MEMORY (mTh1)	FAO	No influence	Low ROS	HIF-1α IKK KEAP-1 NRF-2	CATALASE GSH SOD NADPH	Not described, yet
	EFFECTOR (helper, Th1eff)	GLYCOLYSIS (ANAEROBIC) FAS PPP	Low redox potential ▲ GSH: GSSG				BETULINIC ACID ERYTHRODIOL MARESIN-1 § MOE ^ PROBIOTICS PUFA RESVERATROL SCFA & SFN URSOLIC ACID
	REGULATORY (Treg)	FAO OXPHOS	High redox potential ▼GSH: GSSG	High ROS	FOX-P3 HIF-1α (LOW)	NADP	CURCUMIN ECG EGCG MARESIN-1 § MOE ^ SCFA &

Nutraceuticals showing a pleiotropic activity are marked with unique symbols (§: marensin-1, #: SNF, *: β-sytosterol, ^: MOE, &: SCFA) placed on the right side of the name.

**Table 2:** Nutraceuticals showing anti-diabetic modulatory activities on glucose blood level, oxidative stress, and immune cells responses.

			Anti-Diabetic and Immunomodulatory Activities				
Compound/ Active Component	Natural (Edible) Source	Study Model Cells In Vitro, Animal, Clinical, Epidemiol.	Glycemia	Oxidative Stress	Proinflammatory	Anti-Inflammatory/ Regulatory	References
Epicatechins (ECG, EGCG)	Tea (green, black, others) Leaves	NOD	Hypoglycemic	Antioxidant	NOT DESCRIBED	▼ M1 ▲ M2 ▲ Treg ▲ IL-10	[44,45,46]
Methanolic oregano extract (MOE)	*Origanum vulgare*	NOD	Hypoglycemic	Antioxidant	NOT FOUND	▲ Treg ▲ Th2_EFF_ ▼ Th17 _EFF_	[47]
Polyphenols	*Aronia Melanocarpa* Chockeberry	Human monocytes SZT	Hypoglycemic	Antioxidant	NOT DESCRIBED	▼ NF-κB ▼ IL-6, IL-8, TNF-α ▼ PGE2 ▼ actMo	[48,49,50,51]
DHA/EPA/ARA (Polyunsaturated fatty acids omega-3/-6, PUFAs) (potential therapeutic modality)	Olive oil Fish Cod liver oil Red meat	Cell line Lymphocytes ex vivo TD1 patients DAISY epidemiological study (longitudinal) NOD Hu tumor grafts in mice	▼ T1D Incidence, severity (long-term assumption)	Antioxidant	NOT DESCRIBED	▼ NF-κB ▼ IL-6, IL-8, TNF-α, IFN-γ ▼ PGE2 ▼ M1 ▼ Th1_EFF_ / Th17_EFF_ ▲ IL-10, IL-4 ▼ M2 ▲ Th2_EFF_ ▲ Treg	[40,52,53]
Resveratrol	Grapes Red wine	Cell line NOD	▼ T1D incidence, severity	Antioxidant	NOT DESCRIBED	▼ NF-κB ▼ Th17_EFF_ ▼ TNF-α, IL-6, IL-1β, IL-17 ▲ IL-10	[45,54,55]
Ursolic acid (UA)	Plants Epicuticular wax	SZT	Hypoglycemic	Antioxidant	NOT FOUND	▼ NF-κB ▼ Th1_EFF_ ▼ Th2_EFF_	[21,56]
Berberin (isoquinoline alkaloid)	*Berberis* spp.	Cell line NOD / Clinical Trial (combination treatment with metformin)	Hypoglycemic	Antioxidant	NOT DESCRIBED	Unknown	[57]
Aspalathin	*Aspalathus linearis* Rooibos	Cell line NOD	unknown	Antioxidant	NOT DESCRIBED	▼ NF-κB ▼ TNF-α, IL-6	[58]
Polyphenols, phenolic acids	Coffee beans	Prospective human cohort studies	normal	Weak radical scavenger ▼ T2D risk of disease ▲ Nrf2⟶antioxidant enzymes (+detox, repair)	NOT DESCRIBED	LOW activity	[59]
Isoflavons	Soybeans	Immune cells (cell line) Hu tumor grafts in mice	unknown	Antioxidant	NOT FOUND	▼ NF-κB ▼ IL-6 ▼ M1	[60]
Mangiferin	Mango tree	Hu tumor grafts in mice	unknown	Antioxidant	NOT DESCRIBED	▼ M1 ▼ NF-κB	[61]
Pistacia (oil/hydrosol)	*Pistacia vera/P. lentiscus* Aromatic tree	Cell line	unknown	Antioxidant	NOT FOUND	▼ NF-κB (citrate) ▼ TNFα, IL-6, IL-1β	[62,63]
Saffron	*Crocus sativus*	PBMC ex vivo Hu lymphocytes ex vivo NOD Other murine models	unknown	Antioxidant	NOT FOUND	▲ IL-10 ▲ M2 ▼ TNF-α, IL-6 and IL-1β	[64]
Thymol	*Lippia thymoides* Essential oil	Cell line Hu tumor grafts in mice	unknown	Antioxidant	NOT FOUND	▼ TNFα, IL-6 ▲ Th2_EFF_	[65]
Betulinic acid (pentacyclic triterpene)	*Lycopus lucidus*	Immune cells (cell line) Hu tumor grafts in mice	unknown	unknown	NOT DESCRIBED	▼ TNF-α, IL-2, IFN-γ ▼ Th1_EFF_ ▲ Th2_EFF_	[66]
Erythrodiol (triterpene)	*Humboldtia unijuga*	Immune cells (cell line)	unknown	unknown	NOT DESCRIBED	▼ Th1_EFF_ ▼TNFα, IL-6 and IL-1β ▲ Th2_EFF_	[67]
Maresin 1 (docosahexaenoic acid, DHA-derived)	Macrophage pro-resolving bioactive lipid mediator	Immune cells (cell line) Hu tumor grafts in mice	unknown	unknown	NOT DESCRIBED	▲ M2 ▲ Treg ▲ IL10 ▼ M1 ▼Th1_EFF_/Th2_EFF_/Th17_EFF_	[68]
Oleanolic acid	Medicinal herbs	Immune cells (cell line) NOD	unknown	unknown	NOT DESCRIBED	▲ AMPK	[69]
Squalene (2,6,10,15,19,23-hexamethyl-2,6,10,14,18,20-tetracosahexane)	Virgin olive oil (VOO) (non-saponifiable fraction)	Hu tumor grafts in mice	unknown	unknown	NOT FOUND	▼ M1 ▲ Th2_EFF_ ▲ IL-10, IL-4, IL-13	[70]
Quercetin	Grapes Onion	Cell line NOD	Hypoglycemic	Antioxidant	▲ NK	▼ DC1 ▼ IL-6, IL-1β	[71]
Adenosine-based (non-peptidyl compounds)	Fungi Plants	NOD	unknown	ROS scavengers	NOT DESCRIBED	▲ DCreg ▲ Treg ▲ DC2 ▲ Th2_EFF_ ▼ Th1_EFF_	[72]
*Bacteroides*, *Lactobacilli, Bifidobacteria* and certain *Clostridia*	Probiotics (supplements, food microbic flora)	NOD / Allergic inflam. in BALB/c mice Clinical Study TEDDY	unknown	unknown	NOT FOUND (described for other strains and other inflammatory settings)	▼Insulitis ▼ Th1_EFF_ ▼ Th2_EFF_ ▲ Treg ▲ IL-10 ▼ Eosinophils ▼ Th17_EFF_	[10,37,39]
Acetate Butyrate Propionate (SCFA)	Postbiotics produced by fermentation of indigested carbo by probiotics (supplements, food microbic flora)	7–17 DETC murine cell line (activated proinflammatory innate epidermal γδT cells) Skin NOD Obese mice*	*Butyrate paradox ▲ insulin (resistance) ▲ glucose uptake ▲FAS	▼Glycolisys/ OXPHOS▲ Antioxidant	NOT DESCRIBED	▼ Th1_EFF_ ▼ IFN-γ ▲ Treg ▲ IL-10 ▲CD69 immunoregulatory surface receptor	[73,74,75,76,77]
β-sitosterol	Nuts and other seeds Legumes Virgin olive oil (VOO)	High fat diet + sucrose-induced T2D in rats Hu tumor grafts in mice	Hypoglycemic	Antioxidant	▲ Th1_EFF_	▼ M1 ▲ M2 ▲ Th2_EFF_ ▼ Th2_EFF_	[22,78]
Curcumin	*Curcuma longa*	Hu tumors grafts in mice NOD	unknown	Antioxidant	▲ M1 ▲ Th1_EFF_	▼ M2 ▲ Treg ▲ IL-10 ▼ Th1_EFF_ ▲ Th2_EFF_	[45,79,80]
δ-Oleanolic acid (pentacyclic triterpenoid) Other saponins (adjuvants)	Plants (saponins)	Macrophages Activated T and B cells Splenocytes Hu tumor grafts in mice Clinical trials	unknown	Antioxidant	▲ Th1_EFF_ ▲ Fc receptor ▲ IgA, G1, G2a, G2b ▲ IL-1, IL-2, IL-12	▼ M1 ▼ IL-6, IL-8, TNF-α	[69,81]
Spirulina	*Arthrospira platensis* *Arthrospira maxima* Cyanobacteriaceae (High protein supplement)	Cell line NOD	unknown	Antioxidant	▲ IL-1β, IL-4, and INF-γ	▼TNF-α, IL-6, IL-1β	[82]
Sulphoraphane (SFN)	*Brassicaceae* spp. Vegetables	Hu tumor grafts in mice	Hypoglycemic	unknown	▲ Th1_EFF_	▼ M1 ▲ M2 ▼ Th1_EFF_ ▼ Th2_EFF_	[83]
Vitamin C	Fruits, vegetables (micronutrient)	Immune Cells (cell line)	unknown	Antioxidant	▲ Th1_EFF_ ▲ Neutrophils	unknown	[84]

Each group of natural edible sources of the nutraceuticals is evidenced by a different background. The dark green background addresses the plant-derived nutraceuticals showing all three hypoglycemic, antioxidants, and immunomodulatory activities. The arrows indicate a stimulating (up) or an inhibiting (down) immunoactivity of nutraceuticalsin DM.

## Data Availability

Not applicable.
